# Dental Education Abroad and at Home

**Published:** 1882-02

**Authors:** 


DENTAL EDUCATION ABROAD AND AT HOME.—It is well-known that since the labors of the English Dental Reform Committee resulted in the Registràtion Bill, which gives dentistry in England, as a specialty, a legal status, the dental hospitals have also taken a very important step, i. e. requiring all candidates to pass a preliminary examination.

To-day we learn from *le Progres Dentaire*, that the recently organized *Ecole Dentaire Libre de Paris* has also instituted the *Examin d' Entree*. We quote from its rules:

The preliminary examination shall be passed in the presence of the members of *le Conseil de direction* on the following subjects:

French.—Dictation.

Arithmetic.—Fractions; metric system.

Geography.—Especially of France.

History.—Especially of France.

Elements of geometry.

The candidate may choose for special examination one of the following subjects:

Elements of Physics, Elements of Chemistry, Natural History, Mechanics.

The examination will be both oral and written. The examiners will present a report to the *Conseil de direction*, who will decide as to admissions.

Those candidates will be exempt from this examination who possess one of the baccalaureate degrees.

In other respects the French school will be conducted upon the same general basis as our own, but candor compels us to admit that this preliminary examination of both the English and French schools, as to fitness of the candidate to commence the study of a profession is a step in true progress, worthy of imitation by our own dental colleges, especially so by the universities.

For many years it was our boast that America alone posessed dental schools. Now that our transatlantic confreres have established dental hospitals and schools we fear there is danger that the future may show them to have accomplished more than we have in the direction of elevating dentistry from its present low level, to its true status as a specialty worthy of the best efforts of educated and cultured men.

Of one thing we may rest assured, those schools will not confer degrees upon partial curriculum. Another important feature is that all examinations are made and decisions as to qualifications given, by an official board outside of, and independent of the teachers and professors of the schools.

Prof. Hodgkin, of the Baltimore dental college, begs his southern friends to send better material—students—of which to make dentists. A very pertinent prayer, but let dental schools institute the preliminary examination and better material will be at hand, from which graduates may be developed who shall be a credit to their Alma Mater, and an honor to the specialty.

